# Decoding collaboration obstacles: a survey on reporting preferences between neuroradiologists and clinical specialists

**DOI:** 10.1186/s13244-026-02293-6

**Published:** 2026-05-15

**Authors:** Felix Gunzer, Giovanna Brandi, Reza Seiffert, Thomas Frauenfelder, Zsolt Kulcsar, Till Sprenger, Meritxell Garcia Alzamora

**Affiliations:** 1 Department of Neuroradiology, Clinical Neuroscience Center, University Hospital Zurich, University of Zurich, Zurich, Switzerland; 2 Diagnostic and Interventional Radiology, University Hospital Zurich, University of Zurich, Zurich, Switzerland; 3 Institute for Intensive Care, University Hospital Zurich, University of Zurich, Zurich, Switzerland; 4 Section of Neuroradiology, Department of Radiology and Nuclear Medicine, Neurocenter, Cantonal Hospital Lucerne, University of Lucerne, Lucerne, Switzerland; 5 University Hospital Zurich, University of Zurich, Zurich, Switzerland

**Keywords:** Neuroimaging, Interdisciplinary communication, Communication barriers, Surveys and questionnaires, Report variants

## Abstract

**Objectives:**

To quantify reporting preferences and communication barriers between neuroradiologists and neuro-associated clinicians at a tertiary neuroscience center.

**Materials and methods:**

Two near-identical, multilingual online surveys (17 questions) were distributed to neuroradiologists and clinicians in neurology, neurosurgery, and otorhinolaryngology. The surveys included demographic questions, satisfaction assessments, and report-related preferences. Data analysis was performed using descriptive statistics (frequencies and percentages).

**Results:**

Responses were obtained from 80 physicians (21 neuroradiologists; 59 clinicians). Most clinicians reported reading the entire report (59.3%); subspecialty differences were not significant (chi-square test, *p* = 0.59). In a stroke scenario, context-based structured reports were preferred by 45.8% of clinicians, in contrast to only by 9.5% of neuroradiologists. Standardized classifications and quantitative measurements were endorsed often/always by 59.3%, and key images were considered useful routinely or in complex cases by 89.8% of clinicians. For the clinical neuro-related subspecialties, no significant between-group difference in impact ratings was observed (chi-square test, *p* = 0.25). Neuroradiologists indicated that more detailed clinical information would be beneficial.

**Conclusion:**

Clinicians favor context-based structured reporting with classifications, quantitative measurements, and key images, whereas neuroradiologists predominantly prefer conventional formats. Adopting context-based templates with clear governance may help align expectations and support interdisciplinary workflows.

**Critical relevance statement:**

More focused and detailed clinical information, together with greater use of context-based structured reports incorporating classifications, quantitative measurements, and key images, may facilitate clearer communication between radiologists and clinicians and better support tailored clinical decision-making workflows.

**Key Points:**

- Interdisciplinary reporting needs in neuroradiology were assessed by surveys.
- Clinicians favor context-based structured reports and standardized content.
- Referral quality and report design are modifiable barriers to improving workflow.

**Graphical Abstract:**

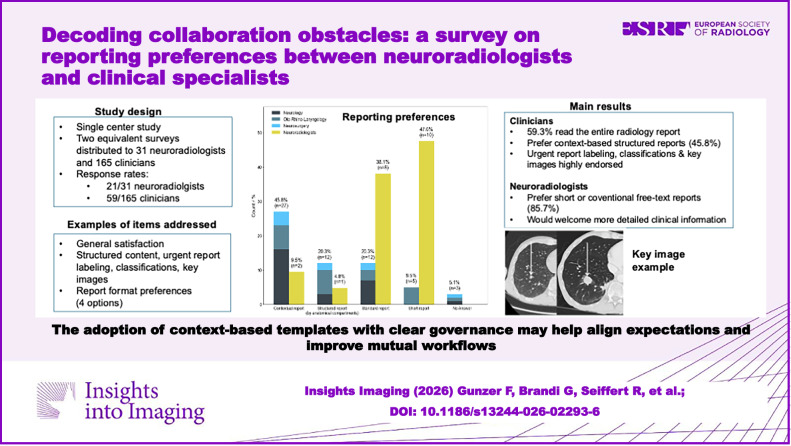

## Introduction

Despite advances in structured reporting (SR), communication barriers between neuroradiologists and referring neuro-associated clinicians appear to still contribute to suboptimal workflows and delays in clinical decision-making [1, 2]. Neuroradiological reports may not fully match clinicians’ needs, while radiologists often receive insufficient clinical context in referral requests [3]. The extent of these preference misalignments and their implications for interdisciplinary collaboration in clinical neurocenters remain insufficiently characterized.

Structured reporting has been advocated to improve clarity and completeness of radiology reports [4, 5], yet its adoption remains limited, partly due to workflow integration challenges and perceived template rigidity [6, 7]. Previous studies have largely focused on general radiology reporting preferences alone, whereas comprehensive data on reporting expectations and communication barriers across neuro-associated clinical disciplines are limited.

In addition, interdisciplinary alignment on reporting-related protocols appears inconsistent between institutions, and formal validation with input from neurology, neurosurgery, and ENT specialists is not routinely established [8]. Clinicians‘ preferences regarding report content, structure, and supplementary elements—such as classifications and key images—have not been systematically assessed in neuroradiology to date.

Therefore, this cross-sectional survey aimed to quantify reporting preferences and communication barriers between neuroradiologists and clinical specialists at a tertiary neurocenter. We compared preferences for report formats, content elements, and communication practices to identify areas of misalignment and to propose potential improvements in neuroradiological reporting.

## Materials and methods

In this prospective, single-center survey study, we investigated reporting preferences between neuroradiologists and referring clinical specialists using comparative analysis of survey responses. Inclusion criteria comprised all physicians (neuroradiology staff and neuro-related clinicians, including neurologists, neurosurgeons, and ENT specialists) actively working at a tertiary neurocenter who participated in the survey. Two nearly identical surveys addressing the respective items from each point of view were developed for neuroradiologists and neuro-associated clinicians, respectively.

**Fig. 1:**
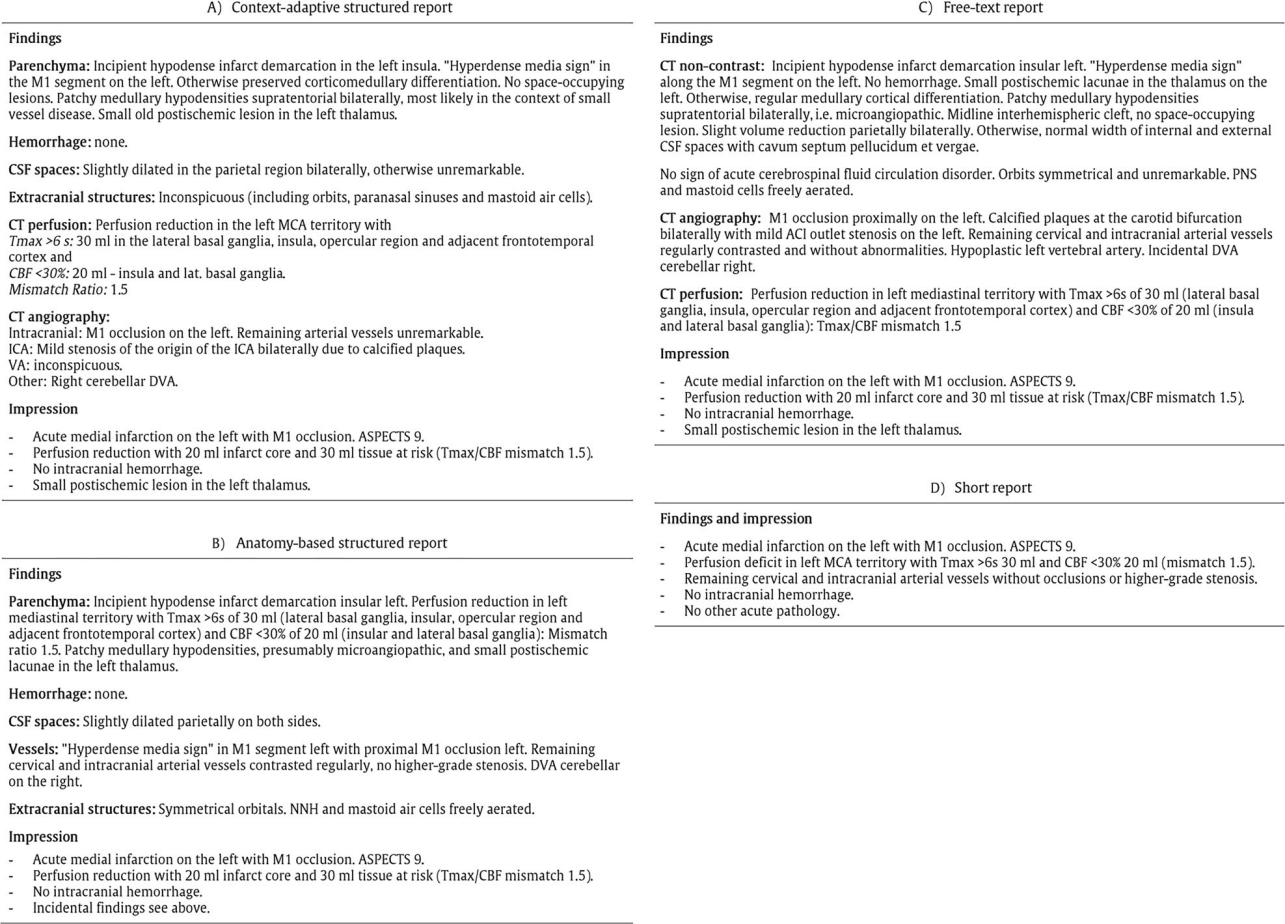
**a** Structured reporting formats: context-based (**A**) and anatomy-based (**B**) SR. **b** Structured reporting formats: free-text (**C**) and short reports (**D**)

Each survey consisted of 17 items, including demographic questions, satisfaction assessments, and report-related preferences using randomized single- and multiple-choice items, conditional follow-ups, and five-point Likert scales (ranging from 0 to 5). The surveys were available in German, French, Italian, and English. Following an informed consent demand, participants answered questions covering satisfaction with interdisciplinary collaboration, current reporting practices, content preferences, and preferred reporting formats. For the latter, they were asked to select one of four exemplary stroke report formats: (1) a context-based structured report, in which key clinical information such as the presence of hemorrhage, parenchymal signs of ischemia, and CT perfusion parameters (e.g., time to maximum (Tmax), cerebral blood flow (CBF), mismatch ratio) is explicitly highlighted and case-dependent accordingly tailored to the specific clinical scenario; (2) an anatomy-based structured report, a standardized format segmented by anatomical compartments (e.g., parenchyma, cerebrospinal fluid (CSF) spaces, vessels); 3) a conventional free-text report, a narrative format without predefined structure allowing full descriptive flexibility; and 4) a short report, consisting of a brief, concise summary of findings and conclusions arranged in few key bullet points (see Fig. 1).

All questions referred to the daily clinical practice, including both elective and emergent settings.

The surveys were distributed to all participants via email using the Typeform platform. The data collection period extended over 5 weeks in 2024, with participant enrollment occurring throughout this timeframe. A single reminder was sent at 3 weeks to maximize response rates. Responses were collected electronically and merged into a German dataset for subsequent analysis.

For the analyses of the clinicians’ and radiologists’ responses, the Python 3.10.1 program was used. Data analysis was partially performed via descriptive statistics illustrating frequencies and percentages of responses, including for reporting format preferences and perceived clinical impact ratings between neuroradiologists and clinicians (see “Results,” section “Preferences in Report Format and Standardization”). Group differences in categorical response distributions were assessed using chi-square tests of independence. For perceived clinical impact, the chi-square test was applied to the distribution of Likert-scale responses to evaluate whether overall response distributions differed between the clinicians and neuroradiologists (see “Results,” section Perceived Impact of Neuroradiological Reports”). Statistical significance was defined as *p* < 0.05 (two-sided).

## Results

### Data of participants

A total of 80 participants were included in the survey, comprising 21 neuroradiologists and 59 clinicians. The cohort consisted of 29 females and 51 males. Age distributions differed between groups: among neuroradiologists 4 (19.0%) were younger than 30 years, 16 (76.2%) were aged 30–45 years, and 1 (4.8%) was older than 45 years, whereas among clinicians 13 (22.0%) were younger than 30 years, 32 (54.2%) were aged 30–45 years, and 14 (23.7%) were older than 45 years. Among the total number of physicians (31 neuroradiologists, 90 neurologists, 30 neurosurgeons, and 45 ENT specialists), the following response rates were obtained: 68% for neuroradiologists, 30% for neurologists and neurosurgeons, and 51% for ENT specialists. The demographic and specialty distributions are presented in more detail in Table 1.

**Table 1:** Characteristics of the survey participants

Age	Under 30	30 to 45	Above 45	
Radiologists	4 (19.0%)	16 (76.2%)	1 (4.8%)	
Clinicians	13 (22.0%)	32 (54.2%)	14 (23.7%)	
Neurosurgery	3 (33.3%)	5 (55.6%)	1 (11.1%)	
ENT	0 (0.0%)	15 (65.2%)	8 (34.8%)	
Neurology	10 (37.0%)	12 (44.4%)	5 (18.5%)	

Sex	Male	Female		
Radiologists	13 (61.9%)	8 (38.1%)		
Clinicians	38 (64.4%)	21 (35.6%)		
Neurosurgery	6 (66.7%)	3 (33.3%)		
ENT	17 (73.9%)	6 (26.1%)		
Neurology	15 (55.6%)	12 (44.4%)		

Level of training	Resident	Deputy senior physician	Specialist	Consultant/chief medical officer
Radiologists	7 (33.3%)	1 (4.8%)	5 (23.8%)	8 (38.1%)
Clinicians	22 (37.3%)	3 (5.1%)	1 (1.7%)	32 (54.2%)
Neurosurgery	4 (44.4%)	2 (22.2%)	0 (0.0%)	3 (33.3%)
ENT	8 (34.8%)	0 (0.0%)	0 (0.0%)	15 (65.2%)
Neurology	11 (40.7%)	1 (3.7%)	1 (3.7%)	14 (51.9%)

Specialty				
Radiologists	Diagnostic Neuroradiology 18 (85.7%)	Interventional Neuroradiology 3 (14.3%)		
Clinicians	Neurology 27 (45.8%)	ENT 23 (39.0%)	Neurosurgery 9 (15.3%)	

*ENT* ear-nose-throat

## Satisfaction assessments and report-related preferences

### Perceived impact of neuroradiological reports

Neuroradiologists predominantly rated their subjective impression of impact of their reports on clinical decision-making as helpful to extremely helpful (score 4–5: 15/21, 71.4%), with 6/21 (28.6%) selecting “extremely helpful” (score 5) and 9/21 (42.9%) “helpful” (score 4); 5/21 (23.8%) chose a neutral rating (score 3) and 1/21 (4.8%) indicated a low impact (score 2). Clinicians similarly reported a strong influence of radiology reports on their clinical decision-making (score 4–5: 44/59, 74.6%), with 11/59 (18.6%) rating “very strong influence” (score 5) and 33/59 (55.9%) “strong influence” (score 4); 10/59 (17.0%) selected moderate influence (scores 2–3), 2/59 (3.4%) indicated minimal influence (score 1), and 3/59 (5.1%) provided no rating, i.e., not applicable/available (N/A). No statistically significant difference in impact-rating distributions was observed between neuroradiologists and clinicians (chi-square test, *p* = 0.25). Subspecialty-specific clinician distributions are shown in Table 2.

**Table 2:** Compact representation of the survey questions and responses of neuroradiologists and clinicians

Survey questions							
The following includes all survey questions directed at clinicians and neuroradiologists, along with the full results of their responses.							
**Question**	**Answers**						
**R: What effect do you think your radiological reports have on your colleagues' clinical decisions?**	**0**	**1**	**2**	**3**	**4**	**5**	**N/A**
	None at all		Neutral			Extremely helpful	
	-	-	1 (4.8%)	5 (23.8%)	9 (42.9%)	6 (28.6%)	-
**C: To what extent do radiology reports influence your clinical decisions?**	**0**	**1**	**2**	**3**	**4**	**5**	**N/A**
	No influence		Moderate influence			Very strong influence	
	-	2 (3.4%)	2 (3.4%)	8 (13.6%)	33 (55.9%)	11 (18.6%)	3 (5.1%)
Neurosurgery	-	1 (11.1%)	1 (11.1%)	2 (22.2%)	1 (11.1%)	4 (44.4%)	-
ENT	-	-	-	3 (13.0%)	19 (82.6%)	-	1 (4.3%)
Neurology	-	1 (3.7%)	1 (3.7%)	3 (11.1%)	13 (48.1%)	7 (25.9%)	2 (7.4%)

C: How do you usually deal with radiological findings in your daily clinical routine?	I usually read the entire report	I only read the impression (summary/conclusion) section	I do not read the report at all
	35 (59.3%)	23 (39.0%)	1 (1.7%)
Neurosurgery	5 (55.6%)	4 (44.4%)	-
ENT	14 (60.9%)	9 (39.1%)	-
Neurology	16 (59.3%)	10 (37.0%)	1 (3.7%)

C: Do you look at the radiological images yourself?	Always	Usually	Rarely	Never	N/A
	37 (62.7%)	20 (33.9%)	1 (1.7%)	1 (1.7%)	-
Neurosurgery	8 (88.9%)	1 (11.1%)	**-**	**-**	**-**
ENT	11 (47.8%)	11 (47.8%)	1 (4.3%)	**-**	**-**
Neurology	18 (66.7%)	8 (29.6%)	**-**	**-**	1 (3.7%)

Survey questions							
Question	Answers						
**R: How satisfied are you with the information (history) and clinical questions provided by your clinical colleagues?**	**0**	**1**	**2**	**3**	**4**	**5**	**N/A**
	Not satisfied		Partially satisfied			Very satisfied	
	2 (9.5%)	2 (9.5%)	8 (38.1%)	6 (28.6%)	2 (9.5%)	-	1 (4.8%)
**R: How often are you disturbed during reporting by work-related matters?** *phone calls from clinicians while reporting*	**0**	**1**	**2**	**3**	**4**	**5**	**N/A**
	0%		50%			100%	
	-	-	3 (14.3%)	6 (28.6%)	9 (42.9%)	3 (14.3%)	-
**C: If you disagree or are sure something was missed in the radiology report, do you contact the responsible person(s)?**	**0**	**1**	**2**	**3**	**4**	**5**	**N/A**
	Never		Sometimes			Always	
	-	4 (6.8%)	5 (8.5%)	14 (23.7%)	16 (27.1)%	20 (33.9%)	-
Neurosurgery	-	1 (11.1%)	1 (11.1%)	4 (44.4%)	1 (11.1%)	2 (22.2%)	-
ENT	-	2 (8.7%)	3 (13.0%)	6 (26.1%)	6 (26.1%)	6 (26.1%)	-
Neurology	-	1 (3.7%)	1 (3.7%)	4 (14.8%)	9 (33.3%)	12 (44.4%)	-

R: Do abbreviations in application forms for a radiological examination cause you trouble?	Always	Often	Sometimes	Rarely	Never	N/A
	1 (4.8%)	5 (23.8%)	12 (57.1%)	2 (9.5%)	1 (4.8%)	-
**C: Do abbreviations in radiological reports cause you trouble**	-	3 (5.1%)	13 (22.0%)	34 (57.6%)	9 (15.3%)	-
Neurosurgery	-	1 (11.1%)	-	7 (77.8%)	1 (11.1%)	**-**
ENT	-	1 (4.3%)	6 (26.1%)	12 (52.2%)	4 (17.4%)	**-**
Neurology	-	1 (3.7%)	7 (25.9%)	15 (55.6%)	4 (14.8%)	**-**

Survey questions							
Question	Answers						
**R: How often do you write classifications/gradings/scores and corresponding measurements in radiological reports?**	**0**	**1**	**2**	**3**	**4**	**5**	**N/A**
	Never		Now and then			Always	
	-	3 (14.3%)	7 (33.3%)	6 (28.6%)	4 (19.0%)	1 (4.8%)	-

C: Would you like to see classifications and measurements in radiology reports?	always	often	sometimes	rarely	never	N/A
	14 (23.7%)	21 (35.6%)	18 (30.5%)	4 (6.8%)	2 (3.4%)	-
Neurosurgery	1 (11.1%)	3 (33.3%)	4 (44.4%)	1 (11.1%)	-	**-**
ENT	5 (21.7%)	8 (34.8%)	5 (21.7%)	3 (13.0%)	2 (8.7%)	**-**
Neurology	8 (29.6%)	10 (37.0%)	9 (33.3%)	-	-	**-**

R: Would you support the integration of reference images (key images) in reports?	Mostly	Only in complex or not-quite-straightforward cases	Never	N/A
	7 (33.3%)	7 (33.3%)	7 (33.3%)	-

C: Would you find key images in the report useful?	Yes, always	Yes, for complex reports	Rather not	Indifferent	N/A
	17 (28.8%)	36 (61.0%)	2 (3.4%)	1 (1.7%)	3 (5.1%)
Neurosurgery	1 (11.1%)	6 (66.7%)	1 (11.1%)	1 (11.1%)	-
ENT	11 (47.8%)	11 (47.8%)	1 (4.3%)	-	-
Neurology	5 (18.5%)	19 (70.4%)	-	-	3 (11.1%)

Survey questions			
Question	Answers		
**C: Would you find an explicit labeling of reports for findings that should be read promptly useful?**	**yes**	**no**	**N/A**
	52 (88.1%)	6 (10.2%)	1 (1.7%)
Neurosurgery	8 (88.9%)	1 (11.1%)	-
ENT	20 (87.0%)	2 (8.7%)	1 (4.3%)
Neurology	24 (88.9%)	3 (11.1%)	-
**R: Does your department have templates for structured findings?**	**yes**	**no**	**N/A**
	9 (42.9%)	11 (52.4%)	1 (4.8%)

R: Does your department use programs supported by artificial intelligence?	Yes	No	I don't know	N/A
	18 (85.7%)	3 (14.3%)	-	-

R & C: Four variants of reports in a case of stroke are shown. Please go through the four report variants and select the one you consider most appropriate.	Contextual report	Structured report (by anatomical compartments)	Standard report	Short report	N/A
Radiologists	2 (9.5%)	1 (4.8%)	8 (38.1%)	10 (47.6%)	-
Clinicians	27 (45.8%)	12 (20.3%)	12 (20.3%)	5 (8.5%)	3 (5.1%)
Neurosurgery	4 (44.4%)	2 (22.2%)	2 (22.2%)	**-**	1 (11.1%)
ENT	7 (30.4%)	7 (30.4%)	3 (13.0%)	5 (21.7%)	1 (4.3%)
Neurology	16 (59.3%)	3 (11.1%)	7 (25.9%)	**-**	1 (3.7%)

Survey questions						
Question	Answers					
**R: Does your institute have a deadline for signing off on elective/non-emergency reports?**	**within 24 h after examination**	**within 48h**	**within 1 week**	**within 2 weeks**	**more than two weeks/no deadline**	**N/A**
	17 (81.0%)	1 (4.8%)	2 (9.5%)	1 (4.8%)	-	-

R: On average, how long does it take you to write an elective/non-emergency radiology report?	<5 min	<10 min	<30 min	<1 h
Xray	16 (76.2%)	3 (14.3%)	2 (9.5%)	-
CT	3 (14.3%)	12 (57.1%)	6 (28.6%)	-
MRI	-	6 (28.6%)	14 (66.7%)	1 (4.8%)
Ultrasound	13 (61.9%)	5 (23.8%)	2 (9.5%)	1 (4.8%)
**C: On average, how long do you wait for an elective/non-emergency radiology report?**	**<1 h**	**>1 to <24 h**	**>24 to <72 h**	**>3 days**
Xray	C: 1 (2.1%) NT: 1 (7.1%)	C: 40 (81.6%) Neurosurgery: 7 (77.8%) ENT: 11 (78.6%) Neurology: 22 (84.6%)	C: 8 (16.3%) Neurosurgery: 2 (22.2%) ENT: 2 (14.3%) Neurology: 4 (15.4%)	-
CT	C: 1 (1.8%) Neurology: 1 (5%)	C: 45 (81.8%) Neurosurgery: 7 (77.8%) ENT: 14 (66.6%) Neurology: 24 (75.7%)	C: 6 (10.9%) Neurosurgery: 1 (11.1%) ENT: 4 (19.1%) Neurology: 1 (5%)	C: 3 (5.5%) ENT: 3 (14.3%)
MRI	-	C: 34 (60.7%) Neurosurgery: 5 (55.6%) ENT: 8 (40.0%) Neurology: 21 (77.8%)	C: 16 (28.7%) Neurosurgery: 4 (44.4%) ENT: 7 (35.0%) Neurology: 5 (18.5%)	C: 6 (10.6%) ENT: 5 (25.0%) Neurology: 1 (11.1%)
Ultrasound	C: 3 (6.4%) ENT: 3 (25.0%)	C: 35 (74.5%) Neurosurgery: 5 (55.6%) ENT: 7 (58.3%) Neurology: 23 (88.5%)	C: 9 (19.1%) Neurosurgery: 4 (44.4%) ENT: 2 (16.7%) Neurology: 3 (11.5%)	-

Survey questions	
Question	Answers
**C: Please indicate any classifications/measurements you would endorse in radiological findings**	Volumetric measurements (tumors, hemorrhages) (20), McDonald criteria (10), hemorrhage severity scores (Fisher score, BNI score) (9), white matter and atrophy assessment (Fazekas scale, Wahlund score [ARWMC], Koedam score) (6), stroke and collateral evaluation (ASPECTS, ASITN/SIR collateral score) (4)

*R* radiologists, *C* clinicians, *ENT* ear-nose-throat specialists, *N/A* no answer

Denominators vary by modality due to multiple selections and non-applicable or missing responses

### How do clinicians approach radiological reports and the corresponding images?

Among clinicians, 35 of 59 (59.3%) reported routinely reading the entire radiology report, while 23 of 59 (39.0%) primarily focused on the impression or conclusion section; only one clinician (1.7%) reported not reading the report at all. Reading behavior did not differ significantly across clinical subspecialties (chi-square test, *p* = 0.59). Independent review of imaging data was common, with 37 of 59 clinicians (62.7%) indicating always and 20 of 59 (33.9%) usually reviewing the radiological images by themselves.

### Satisfaction with clinical information and communication

Neuroradiologists reported overall low satisfaction with the clinical information and referral questions provided by their clinical colleagues. Most respondents (18/21, 85.7%) indicated low to moderate satisfaction (Likert score ≤ 3), while two neuroradiologists (2/21, 9.5%) reported good satisfaction (score 4); no respondent rated their satisfaction as very high (score 5), and one (1/21, 4.8%) provided no rating, i.e., N/A.

Work-related interruptions during reporting (phone calls from clinicians while reporting) were frequent, with 18 of 21 neuroradiologists (85.7%) reporting being disturbed for at least half of their reporting time (score ≥ 3).

In contrast, clinicians frequently sought direct communication with neuroradiologists when disagreeing with radiology reports: 20 of 59 clinicians (33.9%) reported always contacting the responsible neuroradiologists, and an additional 16 of 59 (27.1%) indicated doing so regularly.

### Structured content, classifications, and key images

Clinicians indicated a strong preference for the inclusion of classifications and quantitative measurements in reports: 14/59 (23.7%) selected “always”, 21/59 (35.6%) “often”, 18/59 (30.5%) “sometimes”; 4/59 (6.8%) “rarely”, and only 2/59 (3.4%) “never”. The most frequently endorsed items (multiple responses permitted) were volumetric measurements (tumors/hemorrhages; *n* = 20), McDonald criteria for multiple sclerosis (MS) (*n* = 10), hemorrhage severity scores (e.g., Fisher score and Barrow Neurological Institute (BNI) score; *n* = 9), white matter/atrophy assessment scales (e.g., Fazekas, Wahlund score (ARWMC), and Koedam score; *n* = 6), and stroke/collateral evaluation (e.g., ASPECTS, ASITN/SIR collateral score; *n* = 4).

**Fig. 2:**
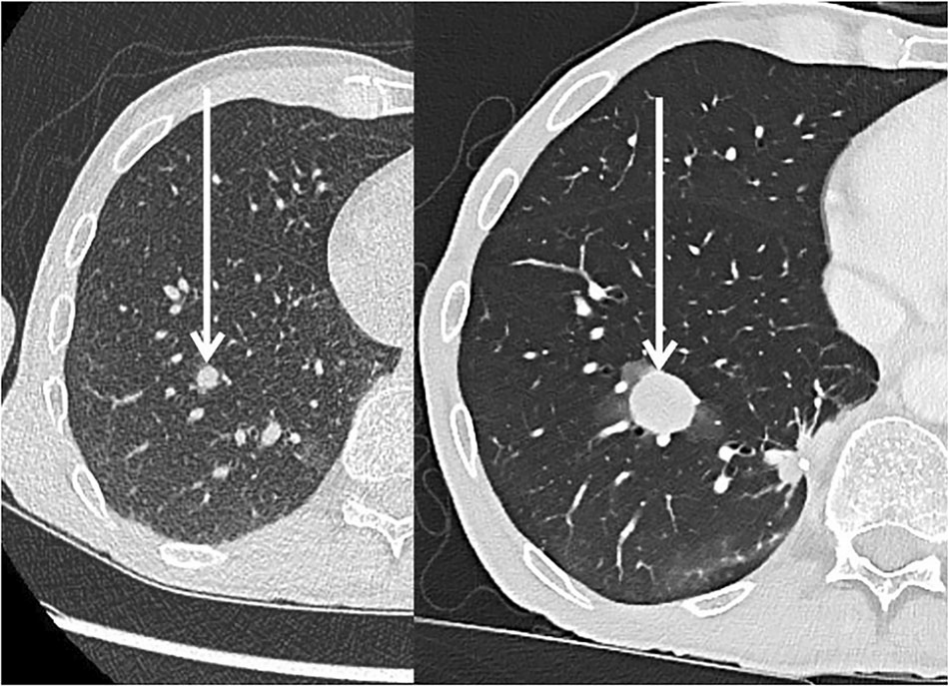
Exemplary key images. Survey background: Perceived usefulness and support for integrating reference (key) images into radiology reports. Clinicians were asked whether they would find key images in reports useful, whereas neuroradiologists were asked whether they would support the integration of key images into reports

The inclusion of reference or key images within reports was also highly valued by clinicians (Fig. 2): 17 of 59 (28.8%) expressed a desire to have them included routinely, while 36 of 59 (61.0%) preferred their use in complex cases only. Among neuroradiologists, 7 of 21 (33.3%) supported the routine inclusion of key images, while 7 of 21 (33.3%) favored their use exclusively in complex or ambiguous cases, and 7 of 21 (33.3%) stated to never integrate key images.

### Preferences in report format and standardization

**Fig. 3:**
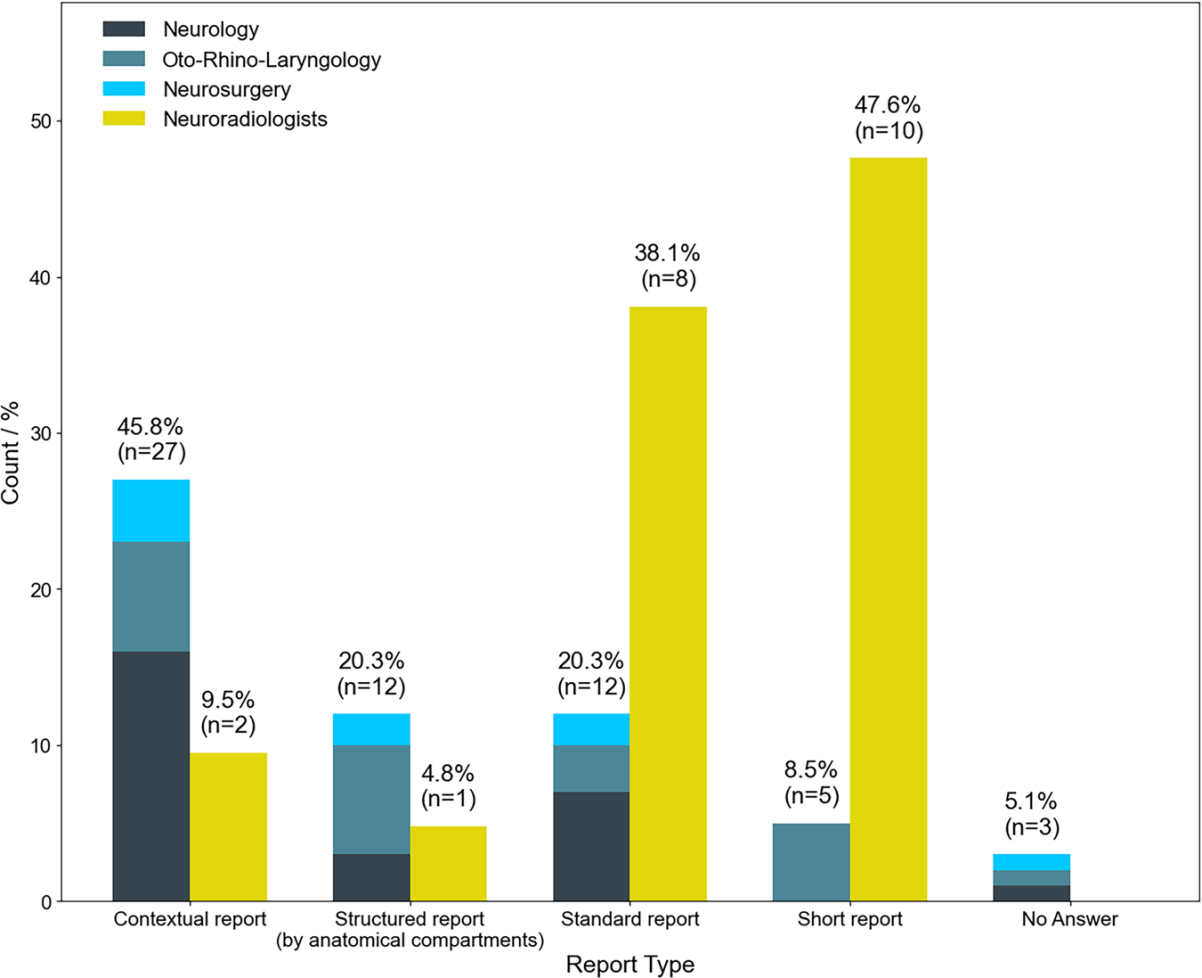
Comparison of preferred report structures among clinical neuros-subspecialists and neuroradiologists

When asked to select a preferred report format in the context of stroke (see Fig. 1), 27 of the 59 clinicians (45.8%) favored context-based structured reports, while only 2 of the 21 neuroradiologists (9.5%) chose this format. The majority of neuroradiologists (85.7%; 18/21) preferred either the short report (47.6%; 10/21) or the standard free-text report (38.1%; 8/21), (Fig. 3).

### Abbreviations as a barrier

Abbreviations were frequently perceived as problematic. Among neuroradiologists, abbreviations in application forms appeared to cause trouble always in 1/21 (4.8%), often in 5/21 (23.8%), sometimes in 12/21 (57.1%), rarely in 2/21 (9.5%), and never in 1/21 (4.8%). Among clinicians, abbreviations in radiology reports were considered problematic, often in 3/59 (5.1%), sometimes in 13/59 (22.0%), rarely in 34/59 (57.6%), and never in 9/59 (15.3%).

### Availability of artificial intelligence (AI)

AI-supported programs were reported to be used by 18/21 neuroradiologists (85.7%), while 3/21 (14.3%) denied the use of AI-supported programs.

### Report turnaround and waiting times

Self-reported reporting times were shortest for X-ray (≤ 10 min: 19/21, 90.5%) and ultrasound (≤ 10 min: 18/21, 85.7%), whereas CT was most commonly < 10 min (12/21, 57.1%) and MRI most commonly < 30 min (14/21, 66.7%; < 1 h: 1/21, 4.8%). Clinician-reported waiting times varied by modality (denominators < 59 due to non-applicable/missing responses): for X-ray (*n* = 49), 1/49 (2.1%) waited < 1 h, 40/49 (81.6%) > 1 to < 24 h, and 8/49 (16.3%) > 24 to < 72 h; for CT (*n* = 55), 1/55 (1.8%) waited < 1 h, 45/55 (81.8%) > 1 to < 24 h, 6/55 (10.9%) > 24 to < 72 h, and 3/55 (5.5%) > 3 days; for MRI (*n* = 56), 34/56 (60.7%) waited > 1 to < 24 h, 16/56 (28.7%) > 24 to < 72 h, and 6/56 (10.6%) > 3 days; and for ultrasound (*n* = 47), 3/47 (6.4%) waited < 1 h, 35/47 (74.5%) > 1 to < 24 h, and 9/47 (19.1%) > 24 to < 72 h.

### Explicit urgent labeling of reports

Clinicians strongly supported explicit labeling of reports containing findings that should be read promptly: 52/59 (88.1%) endorsed such labeling, 6/59 (10.2%) did not, and 1/59 (1.7%) provided no answer.

## Discussion

This cross-sectional survey underscores that neuroradiology reporting is embedded in day-to-day clinical decision-making and that clinicians actively engage themselves in radiology reports rather than treating them as a mere administrative document. Radiology reports are a key interface between disciplines; the best possible mutual adjustment regarding expected demands is of utmost importance [1, 2, 9]. Our data point less toward a fundamental lack of confidence in reporting than toward a design and workflow mismatch at the radiology–clinic boundary.

A central theme is the divergence between the clinicians’ preference for a context-oriented, decision-supportive, and informative architecture and the neuroradiologists’ preference for conventional, flexible formats. This misalignment is consistent with the broader SR literature, which describes two competing needs: (i) standardization and rapid information extraction for downstream care pathways, and (ii) narrative flexibility and efficiency for heterogeneous examinations and complex cases [4, 7, 10, 11]. Contextual or “context-based” reporting approaches have been proposed specifically in neuroradiology to reconcile these priorities by foregrounding the clinically dominant scenario (e.g., hemorrhage vs. ischemia vs. mass lesion) and aligning the report’s structure with clinical reasoning rather than by anatomical compartments or imaging techniques [12]. Importantly, major professional recommendations caution that “structured” should not be equated with rigid, universally standardized templates; instead, successful implementation requires locally governed, use-case-driven rollouts, and iterative refinements with stakeholder inputs [4, 11, 13–15]. Our findings align closely with these recommendations: a workflow-integrated, clinically co-designed approach appears more plausible than the enforcement of an unflexible, broad template.

Preferences for classifications and quantitative measurements highlight the demand for report elements that can be used for prompt comparison with previous numbers and be directly translated into treatment decisions, prognostication, and follow-up planning. The specific items clinicians endorsed map to established neuroimaging decision frameworks (e.g., volumetric assessment, hemorrhage scores, MS criteria, white/gray matter atrophy scales, and stroke/collateral grading), suggesting that clinicians are not primarily asking for “more text,” but rather for structured, comparable, and decision-linked descriptors which can be promptly visualized. The literature supports the view that combining structured report elements with quantitative imaging biomarkers and qualitative scoring systems can improve clarity and completeness, provided that templates remain clinically relevant and feasible to handle in routine practice [10, 16–18]. In this context, the practical challenge is not the conceptual value of standardized metrics, but how to implement them without disproportionately increasing the reporting burden—an issue repeatedly emphasized in implementation studies and position papers [5, 7, 11].

The strong clinicians‘ endorsement of key images and the time labeling of reports (e.g., as urgent) further reflects a preference for report features that support rapid visual impression of the primary finding for quick interpretation and prompt clinical management (accommodating especially for complex cases) as well as for the prioritization of patients.

As most neck and spine examinations include parts of the lungs, we considered the use of a lung nodule as an appropriate key image example for the intended purpose.

Key images can function as a shared visual anchor that reduces ambiguity and aligns mental models across disciplines; however, their utility depends on careful selection, clear linkage to the written conclusion, and governance to avoid medicolegal ambiguity or overreliance on a single snapshot. Professional guidance generally supports such clinically oriented uplifting tools when embedded in a coherent reporting strategy rather than treated as isolated add-ons [11, 19]. Similarly, explicit “read promptly” labeling can improve time-critical communication without the need for a time-consuming specific adaptation or restructuring of the report format, as this step, for instance, can be easily implemented as a small button selection prior to report signing off. Further, such an implementation would align with established safety principles around critical findings communication [8] and may reduce phone calls.

Several workflow and communication barriers emerge as actionable targets that plausibly underlie the preference gap. First, neuroradiologists’ dissatisfaction with referral information is consistent with the broader literature on imaging request quality and its effect on report relevance and efficiency [3]. Context-based reporting requires a reliable clinical premise: sparse, non-specific, or abbreviation-heavy referrals undermine the ability to select appropriate modules, apply correct grading systems, or tailor recommendations. Second, frequent workflow interruptions during reporting are a recognized contributor to cognitive overloading and inefficiency, and may discourage the adoption of more structured, item-rich reporting styles [20, 21]. Improving referral completeness and reducing avoidable interruptions are therefore not mere interventions leading to “more comfort”, but are important prerequisites for implementing the kind of clinically enriched SR clinicians value [22]. Third, the approach of direct contact when disagreement arises can be interpreted in two ways: as evidence of functional accessibility and collaborative culture, or as a signal of residual ambiguity, incomprehension, or even disagreement that might be reduced through clearer structuring, standardized terminology, avoidance of uncertain statements, and provision of selective key images [1, 2, 11, 19].

Turnaround expectation is a further relevant issue. When institutional norms emphasize rapid sign-off and clinicians depend on timely availability, reporting solutions must be realistic under time pressure. The SR literature repeatedly notes that template adoption succeeds when it is tightly integrated into the reporting environment (e.g., smart defaults, automation of repetitive elements, and seamless measurement import) and fails when it is perceived as an “extra step” in terms of “more work associated with time delay” [5, 7, 11], also bearing in mind that some radiologists may be less receptive to workflow changes and prefer conventional full-sentence reporting. From this perspective, quantitative measures and classifications are best framed as workflow-integrated outputs (e.g., automated extraction or structured fields with minimal manual overhead), rather than as manual additions to narrative texts. However, for complex cases, e.g., post-treatment tumor follow-up, pre-defined formats may be disadvantageous if they restrict the needed flexibility to communicate the main findings as clearly as possible. Whether predefined templates may lead to an oversimplification of subtle or benign incidental findings remains to be determined.

Finally, the role of AI and large language models (LLMs) should be positioned conservatively and in line with professional guidance. While LLMs are increasingly explored for drafting, restructuring, or standardizing radiology text, at this stage, the evidence base remains dominated by feasibility studies and retrospective evaluations rather than prospective outcome studies [6, 23–27]. Consistent with ESR recommendations on SR and broader implementation principles, any AI support should be assistive, governed (template versioning, audit trails, validation), and subject to radiologist oversight; otherwise, the risk of plausible but incorrect content (“hallucinations”), subtle distortion of certainties/uncertainties, or inappropriate recommendations may undermine patient safety and trust [6, 11]. In our setting, the reported usage of AI-supported programs suggests a readiness for digital augmentation, but it does not substitute for governance and clinical validation of structured content modules.

Limitations. The findings should be interpreted considering the single-center design with a modest sample size and a relative heterogeneity across clinicians‘ specialties and experience levels, which may limit generalizability.

It could be argued that the data may be biased towards respondents with strong opinions on SR. However, the survey’s title did not explicitly refer to SR but implied an assessment of general collaboration barriers.

The report-format preference task used a stroke example only to keep the survey concise; other neuro-clinical scenarios may yield different preferences. The stroke example was considered appropriate as our institution includes a large vascular center, and SR has not yet been implemented in the Neuroradiology Department, except for MS and neurodegeneration. This may have reduced potential bias related to familiarity with structured templates.

The survey captured perceptions and preferences rather than clinical outcomes; thus, statements about improvements in general efficiency or patient benefit remain unanswered. Finally, some modality-specific workflow questions can yield varying denominators due to non-applicable or unanswered items, which is typical for practice-pattern surveys but limits cross-modal comparability.

## Conclusion

The results support a pragmatic improvement agenda consistent with established recommendations: to co-develop use-case–driven, locally governed context-oriented templates with referring specialties; to strengthen referral information quality to enable meaningful contextualization; to reduce unnecessary reporting interruptions; and to implement selective, high-yield structured elements (e.g., quantitative measures, key images), yet preserving flexibility for complex cases [3, 4, 11, 12, 19, 20]. This approach enables SR to be represented as an adaptable communication framework tailored to the respective workflows.

## Data Availability

The full survey questionnaire is published in this article. Corresponding results are reported in the main manuscript and tables. The anonymized raw survey data are available from the corresponding author upon reasonable request.

## Abbreviations

AI: Artificial intelligence
ARWMC: Age-related white matter changes (Wahlund score)
ASITN/SIR: American Society of Interventional and Therapeutic Neuroradiology/Society of Interventional Radiology (collateral score)
ASPECTS: Alberta Stroke Program Early CT Score
BNI: Barrow Neurological Institute (score)
CBF: Cerebral blood flow
ENT: Ear, nose, and throat
ESR: European Society of Radiology
GPT: Generative pre-trained transformer
LLM: Large language model
MS: Multiple sclerosis
N/A: Not applicable/no answer
SR: Structured reporting
Tmax: Time to maximum
